# Comparing two sampling methods to engage hard-to-reach communities in research priority setting

**DOI:** 10.1186/s12874-016-0242-z

**Published:** 2016-10-28

**Authors:** Melissa A. Valerio, Natalia Rodriguez, Paula Winkler, Jaime Lopez, Meagen Dennison, Yuanyuan Liang, Barbara J. Turner

**Affiliations:** 1 Department of Health Promotion and Behavioral Science, University of Texas School of Public Health in San Antonio, 7411 John Smith Drive, Suite 1100, San Antonio, TX 78229 USA; 2 Center for Research to Advance Community Health (ReACH), University of Texas Health Science Center at San Antonio (UTHSCSA), 7411 John Smith Drive, Suite 1050, San Antonio, TX 78229 USA; 3 South Central Area Health Education Center (AHEC), UTHSCSA, 7411 John Smith Drive, Suite 1050, San Antonio, TX 78229 USA; 4 Frio County AgriLife Extension, 400 S. Pecan Street, Pearsall, TX 78061 USA; 5 Karnes County AgriLife Extension, 115 N. Market Street, Karnes City, TX 78118 USA; 6 Department of Epidemiology and Biostatistics, UTHSCSA, 7703 Floyd Curl Drive, San Antonio, TX 78229 USA; 7 Department of Medicine, UTHSCSA, 7703 Floyd Curl Drive, San Antonio, TX 78229 USA

**Keywords:** Research methods, Sampling studies, Vulnerable populations, Chronic pain, Community-based participatory research

## Abstract

**Background:**

Effective community-partnered and patient-centered outcomes research needs to address community priorities. However, optimal sampling methods to engage stakeholders from hard-to-reach, vulnerable communities to generate research priorities have not been identified.

**Methods:**

In two similar rural, largely Hispanic communities, a community advisory board guided recruitment of stakeholders affected by chronic pain using a different method in each community: 1) snowball sampling, a chain- referral method or 2) purposive sampling to recruit diverse stakeholders. In both communities, three groups of stakeholders attended a series of three facilitated meetings to orient, brainstorm, and prioritize ideas (9 meetings/community). Using mixed methods analysis, we compared stakeholder recruitment and retention as well as priorities from both communities’ stakeholders on mean ratings of their ideas based on importance and feasibility for implementation in their community.

**Results:**

Of 65 eligible stakeholders in one community recruited by snowball sampling, 55 (85 %) consented, 52 (95 %) attended the first meeting, and 36 (65 %) attended all 3 meetings. In the second community, the purposive sampling method was supplemented by convenience sampling to increase recruitment. Of 69 stakeholders recruited by this combined strategy, 62 (90 %) consented, 36 (58 %) attended the first meeting, and 26 (42 %) attended all 3 meetings. Snowball sampling recruited more Hispanics and disabled persons (all *P* < 0.05). Despite differing recruitment strategies, stakeholders from the two communities identified largely similar ideas for research, focusing on non-pharmacologic interventions for management of chronic pain. Ratings on importance and feasibility for community implementation differed only on the importance of massage services (*P* = 0.045) which was higher for the purposive/convenience sampling group and for city improvements/transportation services (*P* = 0.004) which was higher for the snowball sampling group.

**Conclusions:**

In each of the two similar hard-to-reach communities, a community advisory board partnered with researchers to implement a different sampling method to recruit stakeholders. The snowball sampling method achieved greater participation with more Hispanics but also more individuals with disabilities than a purposive-convenience sampling method. However, priorities for research on chronic pain from both stakeholder groups were similar. Although utilizing a snowball sampling method appears to be superior, further research is needed on implementation costs and resources.

**Electronic supplementary material:**

The online version of this article (doi:10.1186/s12874-016-0242-z) contains supplementary material, which is available to authorized users.

## Background

A key feature of community-based and patient-centered outcomes research is partnering with community stakeholders from a project’s inception to ensure that it offers value to the community, is culturally appropriate, and is likely to yield sustainable improvements in prioritized outcomes [1]. Engaging persons from hard-to-reach or vulnerable communities has high priority, given evidence that lack of engaging racial-ethnic minorities and lower socioeconomic populations in research and decision-making contributes to disparities in enrollment in randomized clinical trials, cancer prevention, and access to evidence-based advances in medicine [2–5]. Effective strategies to proactively engage and learn from communities experiencing greater health disparities need to inform the development of community-partnered research [6].

Optimal sampling methods to engage community members to elicit ideas and priorities for community-based participatory research continue to be developed [7, 8]. The Methodology Committee of the Patient-Centered Outcomes Research Institute (PCORI) highlighted improving patient engagement methods as one of four high priority areas for standards development [9, 10]. Although groups experiencing the greatest health disparities likely have the greatest “stake” in ensuring that research meets their personal and community’s needs, well-recognized challenges in establishing partnerships between researchers and members from these communities can undermine efforts to address health disparities [5].

Commonly used sampling methods for identification of participants in community settings vary from random to purposive [11, 12]. It might be expected that researchers should randomly sample from among eligible individuals in a community but this approach is resource-intensive and less demanding sampling methods have performed well in eliciting information that reflects broadly-held community beliefs and ideas [10]. Thus, non-probability sampling methods are preferred for recruitment of stakeholders. Table 1 presents the four sampling methods used most often and some of the challenges and benefits of each one. In addition, ethical challenges can arise in implementing strategies to recruit community partners such as misunderstandings about inclusion and exclusion criteria and potential loss of confidentiality and privacy. However, when conducted in close collaboration with community advisors, engaging representative samples of community members who are most affected by a specific issue such as obesity, HIV, and other issues can yield rigorous relevant research programs [13–15].

**Table 1:** Review of four sampling strategies commonly used in community-engaged research

Sampling method	Definition	Strengths	Limitations	Community engagement and rigor
Purposive Sampling [54, 55]	Strategy allows for selection of a sampling frame that may be most affected by a specific issue.	• Aims to maintain rigor and identify a sampling frame based on specific study driven variables or characteristics.	• Requires collaboration from others to identify participants matching characteristics sought. • Can take time due to specific variables or characteristics sought.	✓✓
Convenience Sampling [10, 56]	Strategy uses existing relationships to identify participants.	• Benefits from existing relationships to identify participants. • Can focus on recruitment from specific locations, settings or activities. • Efficient and inexpensive. • May complete quickly.	• May result in homogeneous sampling frame. • Limited generalizability to broader population. • Less rigorous if organizations or partners do not follow a process to identify participants.	✓✓
Snowball Sampling [10, 29, 57]	Based on a referral approach where a small number of individuals with specific characteristics recruit others with these characteristics from their networks or community.	• Reach to participants with same characteristics. • Often used in community engagement research studies and mixed methods approaches. • Based on networks and relationships which may lend credibility to research.	• Referral contact may not be effective in identifying diverse individuals. • Referral contact may only identify participants meeting specific characteristics. • Participants may not share information freely for fear of privacy or confidentiality – especially in qualitative study.	✓✓✓
Respondent Driven Sampling [30]	Used to reach hidden or most-vulnerable populations basing participation and reach on trust of respondent recruiting frame.	• Seeds recruit a fixed number of participants. • Systematic information collected to identify potential biases.	• Requires training and time to capture and identify respondent relationships. • Reach may not be diverse. • Bias if great percent of participants share characteristics.	✓✓✓

This study was designed to advance understanding of the impact of sampling in community-engaged research by comparing the effectiveness of two non-probability sampling methods to recruit and engage community stakeholders from hard-to-reach, vulnerable populations in identifying priorities for community-partnered research.

## Methods

**Fig. 1:**
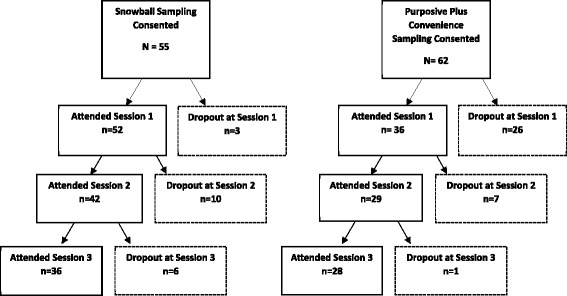
Two Sampling Methods

In two similar rural, predominantly Hispanic counties, one of two sampling methods was selected for use in each community: 1) snowball sampling, a chain-referral method where initial participants (seeds) recruit others from their social network or 2) purposive sampling, also known as judgmental, selective or subjective sampling (Fig. 1). Both methods have been used to recruit hard-to-reach subjects for research studies [16, 17] but have not been compared for the purposes of developing research priorities. For this study, we recruited persons affected by chronic non-cancer pain, either personally or as a caregiver, to generate priorities for research on interventions that could improve outcomes of persons affected by chronic pain in their communities. We conducted a series of three facilitated group meetings, using the nominal group technique, to brainstorm, and prioritize ideas regarding services and support [18, 19] for research on chronic pain.

Our second objective was to compare categories of ideas to improve outcomes of persons with chronic pain generated by participating stakeholders from these two rural, predominantly Hispanic communities. This real-world implementation study not only addresses a priority articulated by PCORI for methods development, it also offers unique insights into priorities for research addressing chronic pain in under-resourced, vulnerable communities.

### Setting

For this project, we partnered with residents of two similar rural communities (Frio and Karnes Counties, Texas) that have had limited involvement in research to advance health despite significant health disparities. These communities are rural and predominantly low income Hispanic. The first community, Frio County, has an estimated population of 18,793 with 70 % of residents aged 19–64 years, 41 % women, and 78 % Hispanic ethnicity (of whom 13 % are foreign born) [20]. Frio County has a median household income of $35,681 and 26 % of residents live in poverty while 68 % completed high school and 23 % are medically uninsured. The second community, Karnes County, has 14,975 residents of whom 70 % are aged 19–64, 41 % women and 52 % Hispanic ethnicity (of whom 6 % are foreign born) [21]. The median household income is $44,650 and 22.3 % of residents live in poverty. Of Karnes residents, 19 % are uninsured, and 72 % have completed high school. Among residents of Frio and Karnes Counties, 12 % and 15 %, respectively, are disabled.

### Study participants

The topic of chronic pain was selected as the community priority to guide the research because of its significant negative impact on health in the U.S., as highlighted in a report by the Institute of Medicine [22]. Residents of the community in both Frio and Karnes were eligible to serve as stakeholders for group meetings about chronic pain if they were 35–75 years of age and experienced chronic pain for at least three months that negatively affected their daily activities and/or sleep. Potential participants were excluded if their pain was due to cancer because of differing management priorities. We also excluded clinicians such as physicians and nurses because as trained experts they may have strong viewpoints and could dominate discussions with patients and caregivers as seen in other studies [23–26]. In addition, caregivers aged 35–75 of persons with chronic non-cancer pain were eligible for participation. In preparation for this project, our research team reviewed several options for non-probability sampling with a standing translational advisory board at our academic institution. Snowball sampling and purposive sampling were selected because the translational advisory board judged them to be likely to yield the desired diverse sample of community members.

### Approach

The project first identified a lead community partner in each community. We successfully engaged the director and staff of Texas A&M AgriLife Extension Services in both counties because this organization shares our mission to improve the health of the community through evidence-based advances. Additionally, a member of our team had previously worked with this group on pilot projects. In each county, the AgriLife Extension agent recruited a community advisory board (CAB) to help develop and guide the study by: reviewing all recruitment materials and methods; assisting with stakeholder recruitment; addressing recruitment problems; and leading meetings to disseminate and act on community stakeholders’ ideas.

#### Sample size

The initial recruitment goal for the study was 130 participants, 65 per sampling method. This goal was based on concept mapping studies where 40 to over 100 participants were involved in idea generation [27].

#### Snowball sampling approach – community 1

Incentivized snowball sampling uses a modified chain-referral approach where a small number of recruits (seeds) meeting eligibility criteria and after consenting to participate then receive a small incentive for recruiting others from their social network who also meet eligibility criteria [28] (Table 2). Persons recruited by “seeds” identified others with desired characteristics and then those individuals identified others until either the sample size goal was achieved or the timeframe for recruitment ended. This approach has the advantage of efficiency and, when networks are broad, final recruits can be independent of initial recruiters [29, 30]. For our project, a convenience sample of “seeds” were first contacted by a CAB member who used talking points developed by the CAB and team members to solicit interest in participation and offered a flyer about the project as additional information. If agreeable, a project coordinator then contacted the individual to assess for eligibility. Eligible “seed” stakeholders (*N* = 12) who consented to participate were informed about the project, provided the talking points developed by the CAB, and asked to recruit others who met study inclusion criteria. The “seeds” received a $5 gift certificate for each eligible recruit.

**Table 2:** Characteristics of stakeholders recruited by sampling method

Characteristic	Snowball sampling stakeholders *N* (%)	Purposive plus convenience sampling stakeholders *N* (%)	*P* Value^a^
Stakeholders	55 (100 %)	62 (100 %)	–
Age, mean ± SD (years)	58 ± 11.97	57 ± 9.77	0.624^c^
Gender			
Female	38 (69)	40 (65)	0.60
Male	17 (31)	22 (35)	
Race/Ethnicity			
Hispanic	48 (87)	45 (73)	0.049
Non-Hispanic White	7 (13)	17 (27)	
Employment Status			
Employed	17 (31)	38 (61)	<0.001
Disability	26 (47)	6 (10)	
Retired	8 (15)	16 (26)	
Unemployed	4 (7)	2 (3)	
Occupation	17 (100 %)	38 (100 %)	0.474^b^
Administrative	1 (6.25)	6 (16)	
Agriculture	2 (6.25)	3 (8)	
Business	2 (12.5)	8 (21)	
Domestic Labor	3 (18.75)	7 (18)	
Education	2 (12.5)	2 (5)	
Government	3 (18.75)	1 (3)	
Healthcare	2 (12.5)	2 (5)	
Manual Labor	2 (12.5)	6 (16)	
Self-employed	0 (0)	3 (8)	
Primary Language			
English	46 (84)	55 (89)	0.089
Spanish	9 (16)	7 (11)	

^a^Chi-Square test

^b^Fisher’s Exact test

^c^Two independent sample *t* test with unequal variances assumption

#### Purposive to convenience sampling approach – community 2

We initially used purposive sampling that identifies participants from specific constituencies from thorough analysis (or database, if available) of the target community’s characteristics and assets [31] (Table 2). Preparation for purposive sampling involved a review of relevant data about each community by CAB members and researchers [20, 21, 32, 33] leading to the development of a matrix of categories of diverse constituencies and gender/age groups to guide recruitment. We aimed to recruit participants with chronic non-cancer pain from diverse community, social, and work organizations including: businesses, volunteer organizations (local Rotary and Lions clubs), faith-based groups, school districts, and agricultural groups such as local ranchers. However, several groups, especially businesses based outside of the community, refused to participate, citing company policies. After conferring with the CAB from community 2, we decided to supplement recruitment with convenience sampling (Table 2). At the CAB’s suggestion, we hired a part-time local businesswoman to assist with recruitment through her small franchise (selling snow cones) that served diverse community members. She was trained in the talking points and provided flyers about the project. After an initial contact, the recruiter then provided data about potential recruits for the research team to evaluate for participation.

### Stakeholder meetings

Within each county, one sampling method was used to recruit three groups of stakeholders to attend a series of three meetings each lasting one to one and a half hours (total of 18 meetings with 9 in each county). All meetings were conducted in Spanish and English and held at convenient times and locations within the same 6 month period. Team members assisted persons with low literacy to understand and participate in activities. The first session provided an orientation about chronic pain and study procedures, including a video of a Hispanic patient describing her experience with chronic pain and a Hispanic primary care physician discussing his approach to managing chronic pain and challenges. The second session was led by an expert facilitator, one per community, and addressed a focused question: “What services or programs are needed to improve the lives of persons with chronic pain?” The participants generated ideas in response to this focused question in a brainstorming meeting structured by the nominal group technique. Developed by social-psychologists, the nominal group technique is the most commonly used structured group method to generate, combine, and prioritize ideas [34]. Initially, participants separately respond to a focused question and then list their ideas “round robin” style. All ideas are reviewed by the group in a facilitated discussion, categorized, and rated. This approach generates diverse ideas within a short timeframe, allowing each individual to contribute instead of only the most outspoken [19]. In the last session, participants grouped ideas into categories and rated separate ideas on both importance and feasibility on five point Likert-type scales. Study protocols were reviewed by the University of Texas Health Science Center at San Antonio Institutional Review Board and determined to be non-regulated research or exempt.

### Analysis

Results were analyzed using qualitative and quantitative methods. To examine differences in demographic characteristics of participating stakeholders in the two counties (e.g., demographic differences between two sampling methods), the chi-square test or Fisher’s Exact test was used for categorical variables and the two independent sample *t* test with unequal variances assumption was used for continuous variables. Because each group of stakeholders sorted their brainstormed ideas into somewhat different groups, three members of the research team independently reviewed and developed categories for the ideas generated by participating stakeholders [35]. Final categories for coding were developed after a discussion of differences among the categories that were previously generated. A final coding of all ideas was conducted by two coders and differences resolved after review by the research team.

A database was created of all community stakeholders’ rankings of their group’s ideas in regard to importance to improve outcomes of persons with chronic pain and feasibility of implementation on a five-point Likert-type scale (i.e., not at all important, somewhat important, very important, extremely important; feasibility scale constructed similarly). For each participant, the mean of their ratings on importance of all ideas within a unique category was calculated and another mean calculated for ratings on feasibility of all ideas within a category. Then these participant-specific mean ratings for each category on each dimension were averaged for all participants within the same community. The mean rating for each category on each of the two dimensions was compared between the two communities using two-sample *t* test with unequal variances assumption. Lastly, the CAB in each community reviewed these results and presented them to community leaders in order to develop strategies and research projects addressing the highest priorities.

## Results

Characteristics of the two groups of stakeholders recruited using two different sampling methods show many similarities (Table 2). The participants recruited by snowball sampling and purposive-convenience sampling were: mean age 58 versus 57 years, 69 versus 65 % women, and 84 versus 89 % preferring English (all *p* > .05). The distribution of occupations also did not differ (*p* = 0.47). However, the snowball sampling strategy had a larger proportion of Hispanic participants than purposive-convenience sampling (87 versus 73 %, respectively, *p* = 0.049) and a larger proportion of participants with a disability (47 versus 10 %, *p* = <0.001).

Using the snowball recruitment strategy, 67 potential participants were contacted, 65 (97 %) were eligible, and 55 (85 %) consented to participate. Of these 55 stakeholders, 52 (95 %) attended the orientation meeting and 36 (65 %) attended all meetings (Fig. 1). In the other community, purposive sampling recruited 21 eligible stakeholders but was supplemented by convenience sampling to increase timely recruitment. Using this combined sampling method, 71 stakeholders were contacted of whom 69 (97 %) were eligible, 62 (90 %) consented, and 36 (58 %) attended. Overall, 26 (42 %) individuals participated in all meetings (Fig. 1). As shown in Table 3, within each community, the same recruitment strategy recruited three separate stakeholder groups who attended the series of three meetings but snowball sampling consistently yielded higher attendance rates to all meetings. Comparison of characteristics of stakeholders who attended the orientation session (Table 4 left columns) reveals that in the first meeting, snowball sampling resulted in a higher proportion of Hispanic participants than purposive-convenience sampling (90 vs. 75 %, respectively *p* = 0.052) as well as persons with disabilities (48 vs. 6 %, *p* < 0.001), respectively. Among participants attending all three sessions (Table 4 right columns), the significant difference in disability status persisted (*p* = 0.006).

**Table 3:** Stakeholder participation by sampling method group^a^

	Snowball sampling stakeholders				Purposive plus convenience sampling stakeholders			
	Group 1	Group 2	Group 3	TOTAL	Group 1	Group 2	Group 3	TOTAL
	*N*			*N* (%)	*N*			*N* (%)
Consented to Participate	17	20	18	55 (100)	21	23	18	62 (100)
Orientation	17	19	16	52 (95)	12	12	12	36 (58)
Brainstorming	16	16	10	42 (76)	10	9	10	29 (47)
Rating/Sorting	11	15	10	36 (65)	8	8	12	28 (45)
Participated in all meetings	11	15	10	36 (65)^b^	8	8	10	26 (42)^b^

^a^In each county, three groups of participants met and each group attended a series of three meetings

^b^Calculated by dividing the number of participants who attended all three meetings by the number of participants consented to participate

**Table 4:** Characteristics of stakeholders within each sampling method group attending first orientation session and three sessions

	Attending first orientation session			Attending all three sessions		
Characteristic	Snowball sampling stakeholders	Purposive plus convenience sampling stakeholders	*P* Value^a^	Snowball sampling stakeholders	Purposive plus convenience sampling stakeholders	*P* Value^a^
	*N* (%)	*N* (%)		*N* (%)	*N* (%)	
Stakeholders	52 (100 %)	36 (100 %)	-	36 (100 %)	28 (100 %)	-
Age, mean ± SD (years)	58 ± 11.62	59 ± 8.73	0.646^c^	59 ± 10.27	59 ± 8.73	>0.999^c^
Gender						
Female	37 (71)	26 (72)	0.913	27 (75)	22 (79)	0.74
Male	15 (29)	10 (28)		9 (25)	6 (21)	
Race/Ethnicity						
Hispanic	47 (90)	27 (75)	0.052	32 (89)	21 (75)	0.144
Non-Hispanic White	5 (10)	9 (25)		4 (11)	7 (25)	
Education						
Less than high school	16 (31)	5 (13)	0.131	12 (33)	5 (18)	0.199
High School or GED	25 (48)	16 (44)		18 (50)	12 (43)	
Post High School	6 (12)	8 (22)		3 (8)	6 (21)	
College Graduate	5 (10)	7 (19)		3 (8)	5 (18)	
Employment Status						
Employed	16 (31)	21 (58)	0.0002	10 (28)	16 (57)	0.006
Disability	25 (48)	2 (6)		16 (44)	2 (7)	
Retired	7 (13)	11 (30)		6 (17)	8 (29)	
Unemployed	4 (8)	2 (6)		4 (11)	2 (7)	
Occupation	16 (100 %)	21 (100 %)		10 (100 %)	16 (100 %)	
Administrative	1 (6.25)	1 (5)	0.706^b^	0 (0)	1 (6)	0.45^b^
Agriculture	1 (6.25)	2 (10)		1 (10)	1 (6)	
Business	2 (12.5)	7 (33)		1 (10)	6 (38)	
Domestic Labor	3 (18.75)	3 (14)		0 (0)	2 (13)	
Education	2 (12.5)	2 (10)		2 (20)	2 (13)	
Government	3 (18.75)	1 (5)		3 (30)	1 (6)	
Healthcare	2 (12.5)	1 (5)		2 (20)	1 (6)	
Manual Labor	2 (12.5)	2 (10)		1 (10)	2 (13)	
Self-employed	0 (0)	2 (10)		0 (0)	0 (0)	
Primary Language						
English	43 (83)	32 (89)	0.421	34 (94)	27 (96)	0.71
Spanish	9 (17)	4 (11)		2 (6)	1 (4)	

^a^Chi-Square test

^b^Fisher’s Exact test

^c^Two independent sample t test with unequal variances assumption

After brainstorming ideas about interventions needed to improve outcomes of community members with chronic pain (Sessions 1–2), stakeholders rated the priority of each idea on a five-point Likert type scale regarding importance and feasibility to implement. The ideas that were generated by each community and their importance rating are included (Additional file 1). Overall, importance ratings were higher than feasibility ratings (Table 5). For six of the eight categories, ratings on importance did not differ significantly between stakeholders recruited by the two sampling methods. Both stakeholder groups rated professional chronic pain support as very important on average. Notably, specific ideas categorized under professional services and support included a variety of non-pharmacologic sources of care such as a physical therapist, nurse counseling, and pain management support regarding mental health and other complications (Additional file 1). Stakeholders recruited by purposive/convenience sampling rated massage therapy significantly higher (diff = −0.35, *p = 0.045*) while stakeholders recruited by snowball sampling rated nutritional programs and city improvements/transportation services for persons with chronic pain more highly but the difference was significant only for the latter (diff = 0.62, *p = 0.004*).

**Table 5:** Importance and feasibility of needed pain management services and support from community stakeholders grouped by recruitment method^a^

Importance rating					Feasibility rating			
Category of services or support needed to improve outcomes of persons with chronic pain	Snowball sampling stakeholders mean (SD)	Purposive plus convenience sampling stakeholders mean (SD)	Diff	*P* Value^b^	Snowball sampling stakeholders mean (SD)	Purposive plus convenience sampling stakeholders mean (SD)	Diff	*P* Value^b^
Professional Chronic Pain Support	4.26 (0.63)	4.04 (0.71)	0.22	0.195	3.89 (0.84)	3.66 (1.01)	0.23	0.324
Nutrition Program	4.16 (0.96)	3.75 (1.14)	0.41	0.124	3.83 (0.86)	3.33 (1.22)	0.50	0.059
Massage Therapy	4.07 (0.78)	4.42 (0.52)	−0.35	*0.045*	3.73 (1.07)	4.00 (0.95)	−0.27	0.297
Education/Outreach	3.90 (0.71)	3.87 (0.93)	0.03	0.884	3.71 (0.82)	3.73 (0.97)	−0.02	0.929
City Improvements/Transportation	3.83 (0.71)	3.21 (0.97)	0.62	*0.004*	3.50 (0.83)	3.23 (1.09)	0.27	0.265
Non-Professional Chronic Pain Support	3.81 (0.76)	3.83 (0.85)	−0.02	0.921	3.71 (0.96)	3.60 (1.00)	0.11	0.657
Water Therapy	3.78 (0.80)	3.86 (1.08)	−0.08	0.735	3.54 (0.91)	3.35 (1.30)	0.19	0.494
Exercise/Fitness Facility	3.71 (0.59)	3.77 (0.86)	−0.06	0.742	3.66 (0.64)	3.54 (0.96)	0.12	0.552

^a^Ordered by priority rating of the Snowball Sampling Group

^b^Two-sample *t* test with unequal variances assumption

None of the ratings on feasibility of implementing these interventions differed significantly between the groups of stakeholders, with most categories rated as being feasible or very feasible. The largest difference in feasibility ratings between the groups was observed for nutritional programs, which was rated as being more feasible by the stakeholders recruited by snowball sampling (diff = 0.50, *p* = 0.059).

## Discussion

In a seminal report, Unequal Treatment, the Institute of Medicine highlighted limited acceptance and involvement in research by community members as a major barrier to sustainable implementation and adoption of health care advances [36, 37]. Engaging community members starting from earliest stages of developing a research project can increase both acceptance and potential sustainability of research results within the community [10, 38]. Community partnerships are especially critical in promoting participation from hard-to-reach populations in all phases of research [39–41]. However, methods to elicit community priorities, especially those of vulnerable, hard-to-reach communities, have been subject to limited evaluation. This study offers valuable insights from implementing two common non-probability sampling methods to recruit individuals from similar rural, predominantly Hispanic communities.

A major finding from this project was that purposive sampling, intended to achieve participation from diverse community constituencies, was challenging to implement largely because of limited cooperation from employers, especially those based outside of the community. In their review of sampling methods to engage stakeholders to identify research priorities, O’Haire and colleagues noted that databases of potential participants are often used for purposive recruitment [10]. For example, purposive sampling has been successfully employed to recruit physicians and leaders within a community [42]; however, at the population level, it is highly unlikely that a purposive sampling framework would be available and, if not, it would require significant effort to build. Because our research team lacked a database for purposive sampling and recruitment was flagging, the CAB in community 2 recommended that we transition to a convenience sampling method, as in other community engagement studies [43]. Convenience sampling was accomplished by hiring a part-time small business owner who interacted with a diverse cross-section of community members on a daily basis. She was trained by the CAB and our research team in recruitment methods and, in a short time, was able to identify potentially eligible low income, Hispanic residents with or affected by chronic pain as caregivers. A pragmatic approach to sampling that combines methods to accommodate challenges such as nonparticipation and inability to locate a target population has been adopted or promoted by others to engage hard-to-reach populations [44–46].

Another key finding was that snowball sampling recruited a larger number of eligible stakeholders. Other studies have found that snowball sampling is particularly effective in hard-to-reach or ‘hidden’ populations because it takes advantage of established social networks of persons with characteristics of interest [47–49]. The CAB in community 1 also served a vital role in operationalizing snowball sampling by identifying the “seeds” who were individuals with or affected by chronic non-cancer pain. This initial recruitment of seeds by the CAB can be regarded as a form of convenience sampling – which in this case resulted in a higher proportion of persons with disabilities with pain, possibly because it was evident to CAB members that these community members suffered from chronic pain. Thus, our snowball sampling method actually integrates initial convenience sampling.

In addition, attendance to all meetings was higher for the snowball sampling recruits than for purposive plus convenience sampling. Promotion of our project by the “seed,” who is a known community member, may have encouraged attendance. However, snowball sampling also recruited more participants who were more severely affected by chronic pain, as manifested by being disabled, compared with those recruited using purposive plus convenience sampling. Thus, careful attention to the characteristics of the seeds and their contacts is needed to promote a balanced representation of stakeholders in the community. Gratifyingly, both sampling methods resulted in recruitment of low-income, predominantly Hispanic community members though snowball sampling recruited a higher proportion of Hispanics.

Despite recruitment with different sampling methods, stakeholders affected by chronic pain in both counties generated relatively similar ideas and priorities for services and support needed to improve outcomes of persons with chronic pain. Professional treatment for chronic pain was rated as very important but this category includes multiple types of professionals delivering non-drug therapies. All community members also gave a high rating to massage therapy but the snowball sampling group in community 1 judged this to be less important, possibly reflecting this group being more disabled. Small studies suggest that the impact of massage on pain or functional outcomes may be diminished for disabled persons [50]. Other priorities included: non-professional support for chronic pain such as group meetings and activities such as arts and crafts; community education about chronic pain; and water therapy. These priorities differ significantly from those of persons with chronic pain who have been treated with long-term opioid analgesics because the latter group focuses primarily on logistics and challenges of obtaining these drugs [51, 52]. These data also suggest that residents of these communities may have a better appreciation of the multi-faceted nature of pain management, in alignment with new national guidelines to use non-opioid drugs and complementary therapies as first line approaches to manage chronic pain [53]. A fertile line of research follows from these community priorities to study implementation and outcomes of evidence-based non-pharmacologic therapy in under-resourced, low income populations.

Limitations of this study include its location – rural Texas counties – that may not be relevant to other low income, predominantly minority communities. However, in support of generalizability to rural Hispanic communities, these two groups independently arrived at relatively similar ideas and priorities regarding services for chronic pain. The study had a relatively small number of participants but our sample size is similar to other stakeholder engagement studies [9]. The robustness of our results may have been increased because we convened three small groups within each county, fostering generation of more ideas through greater opportunities for each stakeholder to contribute. Another limitation relates to the adaptive design of our stakeholder engagement strategy. We transitioned from purposive to convenience sampling to achieve our recruitment goals. However, the similarity of priorities elicited from the two distinct communities supports the value of this combined approach to engage a hard-to-reach population.

The structure of our community partnership also likely contributed to the success of this engagement activity. We had a highly respected lead community partner in each community who spearheaded the establishment of a CAB to guide our stakeholder engagement activities. These CABs now serve as the foundation for ongoing community initiatives to operationalize and conduct research based on topics identified by the stakeholders.

*Implications for Community Engagement-* Implementation of sampling methods to recruit participants for community engaged research need to be guided by community partners. For our study, we relied heavily on two CABs to operationalize different recruitment methods in two similar rural, predominantly Hispanic communities. We found that purposive sampling was challenging to implement due to employers’ lack of cooperation, preventing access to diverse community constituencies; therefore, the CAB directed the research team to transition to convenience sampling. The snowball sampling method was more straightforward to implement and resulted in larger numbers of participants both initially and throughout the series of meetings. Furthermore, it yielded a higher proportion of Hispanic participants whose viewpoints were especially important to solicit in these majority Hispanic communities.

## Conclusions

This study informs methods to engage stakeholders from vulnerable communities to identify research priorities by finding that snowball sampling conducted in partnership with a community advisory board achieved higher attendance rates and greater representation from low income Hispanics. Purposive sampling was more difficult to implement and required guidance from the community advisory board to augment recruitment with a convenience sampling approach. Nevertheless, stakeholders from both communities developed similar research priorities, focusing on diverse non-pharmacologic approaches that are not available in the community to address chronic pain. Future studies need to build on this novel study by examining associated resources and costs for differing sampling strategies to be utilized in hard-to-reach communities that need to be prioritized for research initiatives.

## Additional file

Additional file 1: Importance Rating of Categories of Ideas to Improve Outcomes of Chronic Pain Generated by 2 Sampling Method Groups. Table containing information presented and ranked at the county level of importance and feasibility of interventions for addressing chronic pain within the two communities sampled. (DOCX 24 kb)

## Abbreviations

CAB: Community advisory board
PCORI: Patient-Centered Outcomes Research Institute
