# Supplementary material for: Comparing two sampling methods to engage hard-to-reach communities in research priority setting
Source: BMC Med Res Methodol. 2016 Oct 28;16:146. doi: 10.1186/s12874-016-0242-z (PMC5084459; doi:10.1186/s12874-016-0242-z)
Supplement: Additional file 1: — Importance Rating of Categories of Ideas to Improve Outcomes of Chronic Pain Generated by 2 Sampling Method Groups. Table containing information presented and ranked at the county level of importance and feasibility of interventions for addressing chronic pain within the two communities sampled. (DOCX 24 kb) [file 12874_2016_242_MOESM1_ESM.docx]

| **Importance Rating of Categories of Ideas to Improve Outcomes of Chronic Pain Generated by 2 Sampling Method Groups** | | | | |
| --- | --- | --- | --- | --- |
| Domain, (N Ideas) | Sampling Group^*^ | Statement | Importance Rating† |  |
| **Professional Chronic Pain Support** |  |  |  |  |
|  | 1 |  | **4.23** |  |
|  |  | Some nurse that will explain the good and bad of some medications – belongs in meds | 4.73 |  |
|  |  | Personal care training to train family members who care for terminally ill family members or for the sick | 4.00 |  |
|  |  | Pain management program; to discuss progress, ideas and suggestions | 4.20 |  |
|  |  | Medication class that helps you with your pain | 4.43 |  |
|  |  | Monthly counseling sessions | 4.13 |  |
|  |  | Doctors who are pain specialists | 4.53 |  |
|  |  | Physical Therapy | 4.40 |  |
|  |  | Counseling program to help relieve pain | 4.27 |  |
|  |  | Meditation class | 3.30 |  |
|  |  | Nurse hot line for general info and advice | 4.20 |  |
|  |  | Pain management programs for specific illnesses | 4.29 |  |
|  |  | Physical and occupational therapists | 4.00 |  |
|  |  | Counseling for pain | 4.50 |  |
|  | 2 |  | **4.03** |  |
|  |  | Educational health center nutrition, coping skills, mental health support | 4.13 |  |
|  |  | Help with counseling when you need someone at no expense | 3.88 |  |
|  |  | Monthly support group featuring health consultants | 4.25 |  |
|  |  | Therapy service, program with therapist | 3.88 |  |
|  |  | Local therapy | 4.00 |  |
|  |  | Physical therapist | 4.30 |  |
|  |  | Group facility to talk to doctors about medications | 4.00 |  |
|  |  | Behavior program | 3.38 |  |
|  |  | County nurse to coordinate health related programs that are available | 4.25 |  |
|  |  | Chiropractor, massage, acupuncture center | 3.63 |  |
|  |  | Doctors that deal with holistic approach and osteopathic doctor | 3.88 |  |
|  |  | Counseling | 3.50 |  |
|  |  | County sponsored agency that specializes in pain and movement disorders | 3.88 |  |
|  |  | Rehab center for those with pain | 4.38 |  |
|  |  | Counselor services for depression and anxiety | 4.17 |  |
|  |  | Physical therapy | 4.33 |  |
|  |  | Therapy center | 4.30 |  |
|  |  | Better doctors offices | 4.58 |  |
|  |  | Clinics | 4.25 |  |
|  |  | A center for chronic pain | 4.33 |  |
|  |  | Group therapy | 3.67 |  |
|  |  | Therapy workshop | 3.75 |  |
| **Nutrition Program** |  |  |  |  |
|  | 1 |  | **4.37** |  |
|  |  | Some kind of nutrition program for people that are diabetic | 4.89 |  |
|  |  | Nutritional cooking classes used to have them | 4.64 |  |
|  |  | Dietician to help with healthy eating recipes | 4.80 |  |
|  |  | Dietician program | 4.60 |  |
|  |  | Weekly visits with a nutritionist | 3.57 |  |
|  |  | Nutrition, what to eat and not to eat | 3.53 |  |
|  |  | Nutrition classes tailored for weight loss and pain reduction |  |  |
|  | 2 |  | **3.69** |  |
|  |  | Dietician | 3.83 |  |
|  |  | Nutrition and pain education classes | 4.00 |  |
|  |  | Agency to teach people about food | 3.25 |  |
| **Massage Therapy** |  |  |  |  |
|  | 1 |  | **4.01** |  |
|  |  | Massage therapist home visit to patient with chronic pain. Might not have transportation | 4.27 |  |
|  |  | To have a place for massage therapy | 4.27 |  |
|  |  | Massage therapy that focuses on specific illnesses | 3.93 |  |
|  |  | Massage center | 4.15 |  |
|  |  | Massage therapy for face neck and body | 4.33 |  |
|  |  | Massage class | 4.00 |  |
|  |  | Place to get a massage | 3.50 |  |
|  |  | Acupuncture therapy | 3.64 |  |
|  | 2 |  | **4.42** |  |
|  |  | Massage therapy | 4.42 |  |
| **Education/Outreach** |  |  |  |  |
|  | 1 |  | **3.90** |  |
|  |  | Make video of group with the same situation and the result of the outcome of the individual | 3.00 |  |
|  |  | Therapy training for family members-therapy from family would help everyone | 4.09 |  |
|  |  | Program that familiarizes you with your condition | 4.13 |  |
|  |  | Fellowship, sharing meetings | 3.87 |  |
|  |  | Weekly newsletter about pain management | 4.07 |  |
|  |  | Information related to financial concerns | 3.50 |  |
|  |  | People don’t obey the disabled signs | 4.64 |  |
|  |  | Programs | 3.90 |  |
|  |  | Information meetings | 3.90 |  |
|  |  | Meetings like this to learn | 3.90 |  |
|  | 2 |  | **3.82** |  |
|  |  | Talking about chronic pain | 3.91 |  |
|  |  | Awareness, promote through local schools and businesses | 3.63 |  |
|  |  | County agency to educate people on programs | 4.38 |  |
|  |  | Let’s just see how the community can help and what way they can help when we ask them | 3.63 |  |
|  |  | Health fair events sponsoring chronic pain and other illnesses | 4.63 |  |
|  |  | Have program service available at work, at company | 3.00 |  |
|  |  | Annual event promotion, recognition, funding | 4.00 |  |
|  |  | Workshops | 3.33 |  |
|  |  | Program availability, after work or on my time | 3.88 |  |
| **City Improvements/ Transportation** |  |  |  |  |
|  | 1 |  | **3.87** |  |
|  |  | Leash laws enforced so we can walk on streets | 4.00 |  |
|  |  | Bus with assistance for shopping in San Antonio | 3.36 |  |
|  |  | A way or someone to do yard work-start machine | 3.45 |  |
|  |  | Transportation for those who need it | 4.60 |  |
|  |  | Sidewalks | 4.60 |  |
|  |  | Full service gas stations for pumping gas | 2.60 |  |
|  |  | Curb cuts | 4.00 |  |
|  |  | Equipment rental service to patients with chronic pain | 3.70 |  |
|  |  | Flat sidewalks | 4.00 |  |
|  |  | Day care for pain patients | 4.07 |  |
|  |  | A planting garden, seasonally | 3.40 |  |
|  |  | Walking area for disabled, very well lit | 4.20 |  |
|  |  | In-home care programs, driving, errands, deliveries | 3.60 |  |
|  |  | A well-lit movie theater for disabled | 3.13 |  |
|  |  | House duty, moving, sweeping, mopping, washing clothes, dusting | 3.70 |  |
|  |  | Services to assist people in getting therapy | 4.10 |  |
|  |  | Free medical transportation to appointments and doctors | 4.22 |  |
|  |  | Getting different types of help to assist the elderly at home | 4.00 |  |
|  |  | Services that help people get to the places they need to go | 4.70 |  |
|  | 2 |  | **3.16** |  |
|  |  | Community to help people with medicine for pain when they can’t afford them | 2.88 |  |
|  |  | Provide transportation to and from meetings or pool | 3.00 |  |
|  |  | Try to get volunteers to visit with you. Just because | 3.13 |  |
|  |  | Building where group gathers to do exercise | 3.75 |  |
|  |  | Place to find help | 4.13 |  |
|  |  | Provide transportation | 4.00 |  |
|  |  | Senior center for people with disabilities | 4.38 |  |
|  |  | Hippo therapy center, horses | 2.38 |  |
|  |  | Thrift center for equipment for handicapped (people with disabilities) | 3.13 |  |
|  |  | Equine center, trails and no broncos | 2.25 |  |
|  |  | Garden activities | 2.58 |  |
|  |  | Transportation | 3.13 |  |
| **Non-Professional Chronic Pain Support** |  |  |  |  |
|  | 1 |  | **3.79** |  |
|  |  | Support groups for homebound | 4.09 |  |
|  |  | Support group for people in pain | 4.40 |  |
|  |  | Programs, discussion groups for those with chronic pain | 4.09 |  |
|  |  | Place where we can meet indoors to play bingo | 3.18 |  |
|  |  | Arts and crafts | 3.10 |  |
|  |  | Social support to relieve pain | 4.00 |  |
|  |  | Pain support group | 4.33 |  |
|  |  | Art & crafts activities for movement and to take mind off pain | 4.00 |  |
|  |  | A place to play bingo | 2.87 |  |
|  | 2 |  | **3.86** |  |
|  |  | Meetings continue to discuss pain | 4.75 |  |
|  |  | Meetings and support groups | 4.25 |  |
|  |  | To have a place to share your emotional pain | 3.50 |  |
|  |  | Chronic pain support groups, regular meetings | 4.00 |  |
|  |  | Getting back to work club | 3.63 |  |
|  |  | Talking with others | 3.92 |  |
|  |  | Fun day to get together and vent | 3.75 |  |
|  |  | Group sharing | 3.42 |  |
|  |  | Continue meetings among ourselves | 3.50 |  |
|  |  | Monthly support meeting | 3.88 |  |
| **Water Therapy** |  |  |  |  |
|  | 1 |  | **3.89** |  |
|  |  | Swimming pool, hot tub, exercise equipment, etc. all in one place | 4.18 |  |
|  |  | Swimming and sauna facility for the elderly women only | 3.70 |  |
|  |  | Water aerobics at pool with a way to get in and out of the pool | 3.80 |  |
|  |  | Hot tubs for soaking | 4.30 |  |
|  |  | A place for swimming classes | 3.43 |  |
|  |  | Availability of water therapy in the community | 3.86 |  |
|  |  | Indoor swimming pool for water exercise | 3.90 |  |
|  |  | Pool for therapy | 3.53 |  |
|  |  | Swimming | 4.20 |  |
|  |  | Inside pool | 3.80 |  |
|  |  | Whirlpool to ease the pain | 4.10 |  |
|  | 2 |  | **3.89** |  |
|  |  | Swimming pool | 3.92 |  |
|  |  | Community pool for swimming lessons | 3.75 |  |
|  |  | Aquatic center with water aerobics | 3.63 |  |
|  |  | Health Pool for adults with chronic pain | 4.25 |  |
|  |  | Warm Springs Rehab Center | 4.08 |  |
|  |  | Community pool for therapy | 4.17 |  |
|  |  | Hot tub, muscle relaxer | 4.25 |  |
|  |  | Community Hot Tub | 4.00 |  |
|  |  | Heated exercise pool | 3.75 |  |
|  |  | Swimming pool | 3.88 |  |
|  |  | Spa | 3.92 |  |
|  |  | Hot Tub | 4.08 |  |
|  |  | Sauna | 3.58 |  |
|  |  | Hot tub | 3.38 |  |
|  |  | Pool for exercise | 4.25 |  |
|  |  | Water aerobics | 3.42 |  |
| **Exercise/Fitness Facility** |  |  |  |  |
|  | 1 |  | **3.74** |  |
|  |  | Walking and biking trail indoors or around city pond | 2.73 |  |
|  |  | Obstacle course for physically impaired & patient with chronic pain | 4.09 |  |
|  |  | Exercise programs geared at those with pain at a facility | 4.00 |  |
|  |  | Electronic machine for easy body movements | 3.80 |  |
|  |  | Curves for women | 3.27 |  |
|  |  | A place we can go to exercise | 3.43 |  |
|  |  | YMCA to do exercise | 4.10 |  |
|  |  | Professional therapist instruction for exercise | 4.22 |  |
|  |  | Park to walk | 3.20 |  |
|  |  | A facility to meet all wants and needs | 3.55 |  |
|  |  | To have a place to play tennis | 3.64 |  |
|  |  | Indoor walking lot of bathrooms available | 4.00 |  |
|  |  | Provide exercise programs for homebound | 3.93 |  |
|  |  | Therapist to teach how to stretch | 4.00 |  |
|  |  | Indoor walking track to walk in cool place without heat exhaustion | 3.87 |  |
|  |  | Exercise management with supervision | 4.29 |  |
|  |  | Gym with equipment for therapy | 3.40 |  |
|  |  | Year around exercise classes | 3.90 |  |
|  |  | Yoga classes to assist with stretching | 3.00 |  |
|  |  | Weekly dancing lessons | 4.33 |  |
|  |  | Parks for walking | 4.07 |  |
|  |  | YMCA needed for exercise | 4.00 |  |
|  |  | Move every day | 2.79 |  |
|  |  | Machine workouts, bike or treadmill | 3.80 |  |
|  |  | Walking groups | 3.30 |  |
|  |  | Bike exercise | 3.70 |  |
|  |  | Exercise classes for relieving pain | 4.00 |  |
|  |  | A walking trail | 3.60 |  |
|  |  | Gym | 3.60 |  |
|  |  | Exercise classes | 4.22 |  |
|  |  | Fitness classes | 4.10 |  |
|  |  | YMCA | 4.30 |  |
|  |  | A gym to get therapy | 3.80 |  |
|  |  | Sports Court | 3.00 |  |
|  |  | Running track | 2.60 |  |
|  |  | A place to get therapy | 4.60 |  |
|  |  | Walking Park | 3.90 |  |
|  |  | Exercise gym | 3.90 |  |
|  | 2 |  | **3.77** |  |
|  |  | Wellness center indoor walking, swimming, staffed | 4.25 |  |
|  |  | Donate exercise equipment | 3.25 |  |
|  |  | Appropriate equipment for exercising | 4.50 |  |
|  |  | Inside walking path | 4.13 |  |
|  |  | Accessible exercise facility | 4.38 |  |
|  |  | Make a jogging or walking area | 4.10 |  |
|  |  | Weights | 3.13 |  |
|  |  | Yoga classes with a trained instructor knowledgeable about pain | 3.00 |  |
|  |  | Dance exercise classes low impact | 3.75 |  |
|  |  | Exercise center with classes that target pain-level walking track-sports courts | 3.88 |  |
|  |  | Exercise at our speed | 4.25 |  |
|  |  | Exercise classes for dealing with pain or lack of function | 3.75 |  |
|  |  | Gym trainer for flexibility and stretching exercises | 3.50 |  |
|  |  | Yoga and dance and games | 3.00 |  |
|  |  | Walking path for exercise | 3.75 |  |
|  |  | Exercise places | 4.17 |  |
|  |  | Some easy exercise places | 3.55 |  |
|  |  | Group exercise | 4.00 |  |
|  |  | Exercise center | 4.25 |  |
|  |  | Walking trail | 3.67 |  |
|  |  | Exercise programs | 4.08 |  |
|  |  | Exercises | 3.75 |  |
|  |  | Exercise for adults | 4.33 |  |
|  |  | Sidewalk around the park | 3.75 |  |
|  |  | Wellness center | 3.83 |  |
|  |  | Exercise bikes | 3.38 |  |
|  |  | Tai Chi classes | 2.38 |  |

* 1= Snowball Sampling Group; 2=Purposive and Convenience Sampling Group

† Rating on 5 point Likert-type scale from
